# RIPASA and air scoring systems are superior to alvarado scoring in acute appendicitis: Diagnostic accuracy study

**DOI:** 10.1016/j.amsu.2020.09.029

**Published:** 2020-09-24

**Authors:** Meer M. Chisthi, Anilkumar Surendran, Jiju Therumpurathu Narayanan

**Affiliations:** a Department of General Surgery, Government Medical College, Trivandrum, Kerala, 695011, India; b Department of General Surgery, Government Medical College, Kollam, Kerala, 691574, India; c Department of General Surgery, Government Medical College, Trivandrum, Kerala, 695011, India

**Keywords:** Appendicitis, Appendicitis inflammatory response score, Appendicitis scoring system, Diagnostic test evaluation, Modified Alvarado score, Raja Isteri Pengiran Anak Saleha Appendicitis score

## Abstract

**Background:**

Acute appendicitis remains difficult-to-diagnose in spite of being a common acute abdominal condition. Early and correct diagnosis is essential either to proceed with early appendectomy or conservative approach so that complications and negative explorations can be minimised. Scoring systems can help in quick diagnosis and decision making. Though the Alvarado scoring is the widely used system, differences in diagnostic accuracy have been observed when it is applied to varied populations.

**Materials and methods:**

The objective was to find the predictive accuracy of Modified Alvarado score, Appendicitis Inflammatory Response score and Raja Isteri Pengiran Anak Saleha Appendicitis score, in a diagnostic test evaluation study. From first January 2018 to first January 2019, 107 consecutive patients admitted with a diagnosis of suspected appendicitis were assessed with these scores. Sensitivity, specificity, positive and negative predictive value, positive and negative likelihood ratio and area under curve were determined for each.

**Results:**

Negative appendicectomy rate was 15.89%. Sensitivity, specificity, positive predictive value, negative predictive value and diagnostic accuracy were 64.44%, 58.82%, 89.23%, 23.81% and 63.55% respectively for Modified Alvarado; 97.78%, 29.41%, 88%, 71.43% and 86.92% respectively for Appendicitis Inflammatory Response; 87.78%, 76.47%, 95.18%, 54.17% and 85.98% respectively for Raja Isteri Pengiran Anak Saleha Appendicitis. Area under the curve was 0.726797 for Modified Alvarado, 0.946732 for Appendicitis Inflammatory Response and 0.910131 for Raja Isteri Pengiran Anak Saleha Appendicitis*.*

**Conclusion:**

Appendicitis Inflammatory Response score probably is superior to Alvarado in the paediatric population because the variables scored are easy to apply to children, while Alvarado requires children to identify subjective symptoms which may not always be accurate. Appendicitis Inflammatory Response and Raja Isteri Pengiran Anak Saleha Appendicitis are better diagnostic scoring system for acute appendicitis than Modified Alvarado. Also, both these scores can be easily calculated by complete history, detailed clinical examination and basic laboratory investigations.

## Highlights

• Various scoring systems are used for decision making in appendicitis.
• Modified Alvarado remains the most popular scoring system.
• This study compared Modified Alvarado with Appendicitis Inflammatory Response score and Raja Isteri Pengiran Anak Saleha Appendicitis score systems.
• Sensitivity, specificity and Area under the curve of Appendicitis Inflammatory Response score and Raja Isteri Pengiran Anak Saleha Appendicitis score were better than Modified Alvarado.
• Appendicitis Inflammatory Response score and Raja Isteri Pengiran Anak Saleha Appendicitis scoring system have better accuracy than Modified Alvarado as diagnostic systems.

## 1 Introduction

Acute appendicitis is the one of the commonest reasons for emergency admission to general surgical wards. Acute appendicitis is still a difficult diagnostic entity and the management often involves complex decision making as it involves surgical exploration which utilises technical, financial and human resources. A quick and correct diagnosis of acute appendicitis with subsequent early appendicectomy can avoid complications arising from perforation. Though radiological examinations including Ultrasound and Computed Tomography(CT) scan can further aid in making a definite diagnosis and have been reported to have high sensitivity and specificity, it will inflate the cost to the patient and also the reporting time may further delay emergency appendicectomy. Another worry is regarding the harmful effects of radiation involved in CT scan.

Negative explorations can lead on to longer length of stay in hospital, higher costs and added morbidity and mortality as well. It is accepted that not all cases of appendicitis need to be treated surgically, especially those cases involving catarrhal appendicitis [1]. Unnecessary appendicectomies also should be avoided to avoid potential complications such as ileus (found in 1.2% of cases), incisional hernias (found in 0.68% of cases) and increased cost to the patient [2]. Hence it is beyond doubt that a quick and easy method to diagnose appendicitis in the clinical setting can be of great use to clinicians. With this purpose in mind, various scoring systems have been developed to aid in the clinical diagnosis of acute appendicitis.

Alvarado scoring system, which was first described in 1986, has remained the most popular scoring system in acute appendicitis for many decades. The scoring system remains popular as this scoring system has been proven to have very good sensitivity and specificity [3,4]. The Modified Alvarado Scoring System(MASS) is the system widely used globally. The Appendicitis inflammatory response (AIR) score is a newer scoring system used in suspected appendicitis, first reported in 2008. In previous studies, AIR scoring system has been found to outperform Alvarado scoring system as AIR score utilises more objective symptoms while Alvarado takes more subjective symptoms [5,6]. Also, many studies have independently shown the importance of C-reactive protein(CRP) in the assessment of patients with appendicitis [7,8]. The AIR score has incorporated CRP also as a variable whereas the Alvarado does not.

The Raja Isteri Pengiran Anak Saleha Appendicitis (RIPASA) score is another new diagnostic scoring system developed in 2008 at the Department of Surgery, Raja Isteri Pengiran Anak Saleha Hospital, Darussalam, Brunei. This scoring system, which was initially designed for use exclusively with the Asian population, is broader and simpler and consists of seventeen items with an additional parameter [9]. It has several parameters that are absent in the Alvarado score, such as age, gender and duration of symptoms prior to presentation, which were shown to affect the sensitivity and specificity of Alvarado scoring system in diagnosing acute appendicitis [10].

The three scoring systems, though different in having different maximum scores, have some overlapping parameters [Table 1]. To reiterate the facts, it goes without doubt that any scoring system which can improve over the Alvarado scoring system can turn out to be useful in diagnosing acute appendicitis and thus find generalised acceptance. This study aims to compare the predictive accuracy of AIR score and RIPASA as well as the widely used MASS in diagnosing acute appendicitis by comparing them with the gold standard of histopathologically confirmed appendicitis.

**Table 1:** Comparison of variables used in scoring systems used in appendicitis.

MASS		AIR				RIPASA	
Features	Score	Features			Score	Features	Score
						Patients:	
						Female	0.5
						Male	1.0
						Age <39.9 years	1.0
						Age >40 years	0.5
**Symptoms**		**Symptoms**				**Symptoms**	
Migration of pain	1					Pain Migration to RIF	0.5
Anorexia	1					Anorexia	1.0
Nausea	1	Vomiting			1	Nausea & Vomiting	1.0
		RIF pain			1	RIF pain	0.5
						Duration of Symptoms <48 h.	1.0
						Duration of Symptoms >48 h.	0.5
**Signs**		**Signs**				**Signs**	
Tenderness RLQ	2					Tenderness RIF	1.0
Rebound tenderness	1	Rebound tenderness	Light		1	Rebound tenderness	1.0
		Rebound tenderness	Moderate		2		
		Rebound tenderness	Strong		3		
Elevated temperature	1	Temperature 38.5^0^ C or more			1	Fever >37 °C < 39 °C	1.0
						Guarding	2.0
						Rovsing Sign	2.0
**Investigation**		**Investigation**				**Investigation**	
Leucocytosis	2	White Cell Count (10^9^/l)		10–14.9	1	Raised WBC	1.0
		White Cell Count (10^9^/l)		15 or more	2		
		Proportion of PMNs (%)		70–84	1		
		Proportion of PMNs (%)		85 or more	2		
		C- reactive protein (mg/l)		10–49	1		
		C- reactive protein (mg/l)		50 or more	2		
						Negative Urine Analysis	1.0
Total score	9	Total score			12	Total score	16.5

## 2 Materials and Methods

The primary objective of the study was to estimate the predictive accuracy of Alvarado score and AIR score and RIPASA score against the reference standard of histopathology in patients undergoing emergency appendicectomy at the General Surgical wards of our institution. The current study was designed as a prospective diagnostic test evaluation and carried out for a period of 1 year from January 1, 2018 to January 1, 2019.

Patients undergoing emergency appendicectomy for suspected appendicitis, defined as acute (lasting less than 4 days) non traumatic right iliac fossa pain consistent with a diagnosis of appendicitis (pain associated with nausea, anorexia, vomiting and fever along with clinical signs as tenderness and rebound tenderness in right iliac fossa, with or without ultrasound findings suggestive of appendicitis), were taken as the study subjects. Pregnant females, patients with a right iliac fossa mass, patients with a previous history of urolithiasis or pelvic inflammatory disease, and children below 12 years of age were excluded from the study. Institutional Review committee as well as Ethics committee clearance was obtained before commencing the study. The patients were briefed about the study and signed informed consent obtained before blood sample collection.

Sample size was estimated using standardised formula for sample size estimation in diagnostic test studies, where, sensitivity of the new tests was taken from reference studies [11,12]. Sensitivity of the reference test, that is histopathology was set as 100. With a power of 80% and alpha error of 5%, sample size was calculated for Alvarado, AIR and RIPASA scores separately and the highest value among the three, of 107, was taken as the study sample size.

A score of 7 was taken as high probability of acute appendicitis for Alvarado scoring system and a score of 5 for AIR and 7.5 for RIPASA, as per available literature. All the scores were done only for the study purpose and did not affect management. A detailed questionnaire was made to include the patients' clinical details including presenting symptoms, examination findings and other investigation results. The patients were monitored from the day of admission until discharge from the hospital. Daily follow-up included the monitoring of vital signs and systemic examination. Histopathology findings on the operated cases were collected and correlated with the scores.

The study is reported in line with the STAndards for the Reporting of Diagnostic accuracy studies(STARD) criteria and the checklist included [13]. Data are reported as mean, standard deviation (SD), median (range) or percentage. True positives, true negatives, false positives and false negatives were found out and 2 × 2 tables constructed to determine the sensitivity, specificity, positive and negative predictive values. The correlation between the three scores was tested with Pearson's correlation. The area under the receiver operating characteristic (ROC) curves was used to examine the performance characteristics of the scoring systems individually. The optimal cut off values for attaining maximum sensitivity and specificity were also calculated for the three systems. Statistical analysis was done with Microsoft Office Excel, MedCalc version 19.2(MedCalc Software Ltd) and easyROC software ver 1.3.1 [14]. Significance is reported wherever p < 0.05.

## 3 Results

In the study, there were 60 males(56%) and 47 females(44%) and there were no third gender patients. The mean age of the patients was 25.89( ±1.41). The youngest patient was 13 and the oldest was 70 years old. The overall negative appendectomy rate was 15.89%(17 patients). The Alvarado scores ranged from 4 to 9 with a mean value of 7.33( ±2.12). The AIR scores ranged from 5 to 11 with a mean value of 8.53( ±2.83). The RIPASA scores ranged from 5 to 12 with a mean value of 8.91( ±2.83).

The RIPASA and Alvarado scores were found to be strongly correlated positively, with a Pearson's coefficient of 0.77. The RIPASA and AIR scores were found to be weakly correlated positively, with a Pearson's coefficient of 0.66. The AIR and Alvarado scores were found to be have very weak correlation, with a Pearson's coefficient of 0.54.

Alvarado score was found to have a sensitivity of 64.44%, specificity of 58.82%, positive likelihood ratio of 1.57, negative likelihood ratio of 0.6, positive predictive value of 89.23%, negative predictive value of 23.81% and overall accuracy of 63.55%. The Youden index was calculated to be 0.365. AIR score was found to have a sensitivity of 97.78%, specificity of 29.41%, positive likelihood ratio of 1.39, negative likelihood ratio of 0.08, positive predictive value of 88.00%, negative predictive value of 71.43% and overall accuracy of 86.92%. Youden index was calculated to be 0.8678. RIPASA score was found to have a sensitivity of 87.78%, specificity of 76.47%, positive likelihood ratio of 3.73, negative likelihood ratio of 0.16, positive predictive value of 95.18%, negative predictive value of 54.17% and overall accuracy of 85.98%. Youden index was calculated to be 0.709.

**Fig. 1:**
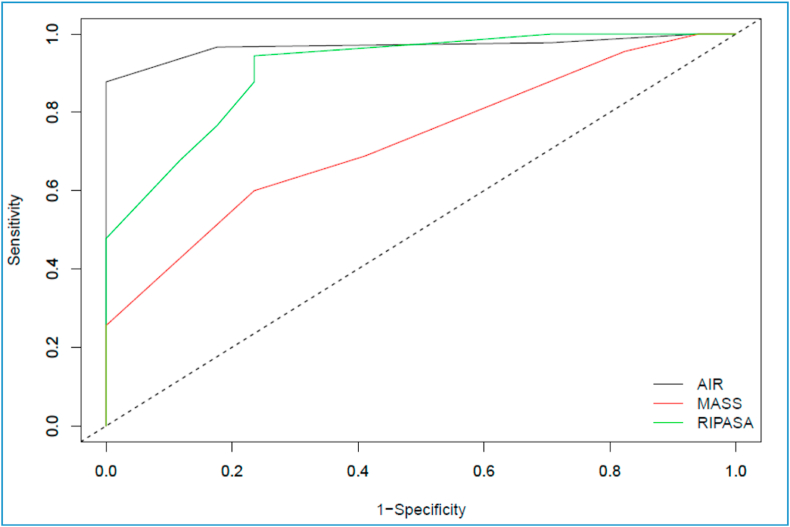
Area Under Curve plots for scoring systems.

The ROC curves were assessed and AUC was estimated. For Alvarado, the AUC was 0.726797, while the AUC for AIR was 0.946732 and the AUC for RIPASA was 0.910131 [Table 2, Fig. 1]. The optimal cut off for achieving maximum sensitivity and specificity for MASS was calculated to be 8, that for AIR was calculated to be 8 and that for RIPASA to be 7.5 [Table 3].

**Table 2:** Comparison of calculated diagnostic values for scoring systems.

Calculated Value	MASS Cut off 7	AIR Cut off 5	RIPASA Cut off 7.5
Sensitivity	64.44%	97.78%	94.4%
Specificity	58.82%	29.41%	76.5%
Positive Likelihood Ratio	1.57	1.39	4.01
Negative Likelihood Ratio	0.6	0.08	0.07
Positive Predictive Value	89.23%	88%	95.5%
Negative Predictive Value	23.81%	71.43%	72.2%
Area Under Curve	0.72679	0.94673	0.91013

**Table 3:** Optimal Cut off values for maximum sensitivity and specificity for scoring systems.

Calculated Value	MASS Cut off 8	AIR Cut off 8	RIPASA Cut off 7.5
Sensitivity	60.0%	87.8%	94.4%
Specificity	76.5%	100%	76.5%
Positive Likelihood Ratio	2.550	Inf	4.01
Negative Likelihood Ratio	0.523	0.122	0.07
Positive Predictive Value	93.1%	100%	95.5%
Negative Predictive Value	26.5%	60.7%	72.2%
Area Under Curve	0.7268	0.96895	0.91013

## 4 Discussion

The overall negative appendectomy rate in the study was 15.89%, which was comparable and lower than those of similar studies [15,16]. A study performed in 2005 in the Netherlands found that approximately 15% of the patients underwent a negative appendectomy, and the number was found to be similar to another large Swedish study [17]. The negative appendectomy rate was as low as 13% in another large volume North American study [18]. However, studies by Rathod et al. [16] and Chong et al. [19] documented higher negative exploration rates of 22.9% and 20.69%, respectively. Large population based studies have suggested that the rate of negative appendicectomies is remaining stable (15–20%) and has not declined for the past 15 years despite the increasing availability of newer tests [20].

Alvarado scoring system has been the most popular scoring system in acute appendicitis for a long time, due to its claimed high sensitivity and specificity [3,4]. The caveat is that the diagnostic efficacy of Alvarado has been well proven in western population only whereas it showed relatively less specificity and sensitivity when applied to oriental populations [21,22]. As per the findings in this study, Alvarado score was found to have medium sensitivity and specificity only. In a study by Memon et al. in Indian population, the sensitivity and the specificity of the Alvarado scoring system were found to be 93.5% and 80.6%, respectively [23]. However, evaluation of Alvarado in a study conducted by Schneider et al. on paediatric population revealed a PPV of 58% only [24]. A systematic review showed that the Alvarado score accurately predicts appendicitis and performs well as a ‘rule out’ criterion for decision making for observation or admission, due to its high sensitivity [25]. However, the review also found that the Alvarado score cannot be used to ‘rule in’ a diagnosis of appendicitis, without proper surgical assessment and further diagnostic testing. The World Society of Emergency Surgeons'(WSES) Jerusalem guidelines in 2015 also stated that the Alvarado score (with cut-off score < 5) is sufficiently sensitive to exclude acute appendicitis but is not sufficiently specific in diagnosing acute appendicitis [26].

The current findings on AIR score of very high sensitivity and low specificity are in line with similar studies. In the study by Scott et al., an AIR score of 5 or more demonstrated high sensitivities for intermediate and high risk patients with appendicitis (90%) and also for patients with advanced appendicitis (98%) [27]. In another study, the AIR score has shown far better results than the Alvarado score [17]. The AIR score probably works better in the paediatric population than the Alvarado score because the variables scored are easy to apply to children. The Alvarado score requires children to identify nausea, anorexia, and migration of pain, which may not always be accurate. Probably this is why the Alvarado score compares better to the AIR score in the adolescent age group, because this age group closely mimics the cohort on which the Alvarado score was first designed. Di Saverio et al. suggested that the combination of AIR and Alvarado scores might significantly reduce the risk of over-diagnosing acute appendicitis and thus give a reliable diagnostic performance, thus enable the treating surgeons to avoid the routine use of CT [28].

The study results found high sensitivity as well as specificity for RIPASA. These are comparable with the study done by Chong et al. [19]. In that study, the RIPASA score at a cut-off threshold total score of 7.5 was found to be a better diagnostic scoring system than Alvarado score for the diagnosis of appendicitis. Rathod et al. obtained a sensitivity of 82.61% and a specificity of 88.89% with the RIPASA score, as well as a PPV of 96.61%, an NPV of 57.14% and a diagnostic accuracy of 83.91% [16]. Nanjundaiah et al. also showed better efficacy for RIPASA over Alvarado in their study [29]. Another study showed a sensitivity level of 81% for the Alvarado system when the cut-off value was set at 6.5, and a sensitivity level of 83.1% for the RIPASA system when the cut-off value was set at 10.25 [12]. On the other hand, there are some studies in which RIPASA score was able to show no advantages over the modified Alvarado score in suspected acute appendicitis [30].

Ohmann and Eskelinen are few of the other scoring systems used for diagnosis of appendicitis in various centres. There are also reports on other diagnostic markers for appendicitis. For instance, a study, based on the results of univariate analyses, found some blood cell surface markers to be useful in the prediction of acute appendicitis namely HLADR + CD19, α/β TCR, and CD3/RA [31]. As per the results of another study, three factors, namely, body temperature ≥37.4 °C, C-reactive protein ≥4.7 mg/dl, and fluid collection surrounding the appendix on CT scan, have been found to be useful in predicting cases of complicated appendicitis preoperatively and facilitate decisions regarding emergency appendicectomy [32].

To summarise, the area under the ROC curve for the RIPASA and AIR scoring systems was significantly larger than it was with the Alvarado system. The RIPASA and AIR scores are fast and are definitely better in categorizing patients with suspected appendicitis and reduce the need for diagnostic imaging. Overall, a higher sensitivity, NPV and PLR and a lower NLR indicate that the RIPASA score and AIR scores are much better diagnostic tools than Alvarado score for diagnosing acute appendicitis in Asian population. The specificity of MASS can be improved significantly with only a minor drop in sensitivity if the cut off is raised to 7.5. However, the overall diagnostic accuracy would remain the same. In the case of AIR, specificity can be hiked to 100% with a slight gain in diagnostic accuracy if the cut off is raised to 8, albeit with a significant drop in sensitivity. For RIPASA, the ideal cut off remains the same at 7.5.

This study is not without its own drawbacks. First, the clinical diagnosis of acute appendicitis in the sample population was based on the clinical judgment of the surgical resident and registrar on duty which could have subjective variations. In addition, patients may have difficulty in defining the time of onset of symptoms. Also, different diagnostic modalities (abdominal ultrasonography) used in selected patients in the department could have affected the negative appendectomy rates detected in the study. Last, the sample size is comparatively small, which could attenuate the significance of the associations.

## 5 Conclusions

An ideal scoring system should work as a tool that speeds up as well as enhances the accuracy of decision-making, and at the same time saves up on the need for expensive or potentially harmful investigations. The Appendicitis Inflammatory Response score probably works better in the paediatric population than the Alvarado score because the variables scored are easy to apply to children. The Alvarado score requires children to identify nausea, anorexia, and migration of pain, which may not always be accurate. To conclude, this study validates that the Appendicitis Inflammatory Response score and Raja Isteri Pengiran Anak Saleha Appendicitis score have high discriminating powers and outperform the Modified Alvarado score. They could aid in selecting patients who require timely surgery or those who require further evaluation. Both these scores have the potential to turn out into scoring systems of choice if future research can substantiate our study findings.

## Funding

This research did not receive any specific grant from funding agencies in the public, commercial, or any other sectors.

## Provenance and peer review

Not commissioned, externally peer reviewed.

## Declaration of competing interest

None.

## Ethical approval

Research studies involving patients require ethical approval. Please state whether approval has been given, name the relevant ethics committee and the state the reference number for their judgement.

Yes.

Human Ethics Committee, Medical College, Thiruvananthapuram.

IEC No.07/15/2017/MCT. Dated July 07, 2017.

## CRediT authorship contribution statement

**Meer M. Chisthi:** Conceptualization, Formal analysis, Writing - original draft. **Anilkumar Surendran:** Conceptualization, Methodology, Writing - review & editing. **Jiju Therumpurathu Narayanan:** Formal analysis, Investigation, Visualization.

## References

[1] Temple C.L., Huchcroft S.A., Temple W.J. (1995). The natural history of appendicitis in adults. A prospective study. Ann. Surg..

[2] Schwerk W.B., Wichtrup B., Rothmund M., Ruschoff J. (1989). Ultrasonography in the diagnosis of acute appendicitis: a prospective study. Gastroenterology.

[3] Alvarado A. (1986). A practical score for the early diagnosis of acute appendicitis. Ann. Emerg. Med..

[4] Owen T.D., Williams H., Stiff G., Jenkinson L.R., Rees B.I. (1992). Evaluation of the Alvarado score in acute appendicitis. J. R. Soc. Med..

[5] Andersson M., Andersson R.E. (2008). The appendicitis inflammatory response score: a tool for the diagnosis of acute appendicitis that outperforms the Alvarado score. World J. Surg..

[6] Sammalkorpi H.E., Mentula P., Leppäniemi A. (2014). A new adult appendicitis score improves diagnostic accuracy of acute appendicitis - a prospective study. BMC Gastroenterol..

[7] Moon H.M., Park B.S., Moon D.J. (2011). Diagnostic value of C-reactive protein in complicated appendicitis. J Korean Soc Coloproctol.

[8] Andersson R.E.B. (2004). Meta-analysis of the clinical and laboratory diagnosis of appendicitis. Br. J. Surg..

[9] Butt M.Q., Chatha S.S., Ghumman A.Q., Farooq M. (2014). RIPASA score: a new diagnostic score for diagnosis of acute appendicitis. J Coll Physicians Surg Pak.

[10] Wani M.M., Yousaf M.N., Khan M.A. (2007). Usefulness of the Alvarado scoring system with respect to age, sex and time of presentation, with regression analysis of individual parameters. Internet J. Surg..

[11] de Castro S.M.M., Unlu C., Steller E.P. (2012). Evaluation of the appendicitis inflammatory response score for patients with acute appendicitis. World J. Surg..

[12] Erdem H. (2013). Alvarado, Eskelinen, Ohhmann and Raja Isteri Pengiran Anak Saleha appendicitis scores for diagnosis of acute appendicitis. World J. Gastroenterol..

[13] Bossuyt PM, Reitsma JB, Bruns DE, Gatsonis CA, Glasziou PP, Irwig L, LijmerJG Moher D, Rennie D, de Vet HCW, Kressel HY, Rifai N, Golub RM, Altman DG, Hooft L, Korevaar DA, Cohen JF, For the STARD Group. STARD 2015: An Updated List of Essential Items for Reporting Diagnostic Accuracy Studies.

[14] Goksuluk D., Korkmaz S., Zararsiz G., Karaağaoğlu A.E. (2016). easyROC: an interactive web-tool for ROC curve analysis using R language environment. The R Journal.

[15] Malyar A.A., Singh B., Dar H.M., Ahmad M.M., Bhat S.B. (2015). A comparative study of appendicitis inflammatory response (AIR) score with Alvarado score in diagnosis of acute appendicitis. Balkan Military Med. Rev..

[16] Rathod S., Ali I., Bawa A.P., Singh G., Mishra S., Nongmaithem M. (2010). Development of the RIPASA score: a new appendicitis scoring system for the diagnosis of acute appendicitis. Singap. Med. J..

[17] Andersson R.E., Hugander A., Thulin A.J. (1992 Jan). Diagnostic accuracy and perforation rate in appendicitis: association with age and sex of the patient and with appendicectomy rate. The European Journal of Surgery = Acta Chirurgica.

[18] Hale D.A., Molloy M., Pearl R.H., Schutt D.C., Jaques D.P. (1997). Appendectomy: a contemporary appraisal. Ann. Surg..

[19] Chong C.F., Thien A., Mackie A.J.A. (2011). Comparison of RIPASA and Alvarado scores for the diagnosis of acute appendicitis. Singap. Med. J..

[20] Flum D.R., Morris A., Koepsell T., Dellinger E.P. (2001). Has misdiagnosis of appendicitis decreased over time? A population-based analysis. J. Am. Med. Assoc..

[21] Jang S.O., Kim B.S., Moon D.J. (2008). Application of Alvarado score in patients with suspected appendicitis. Korean J. Gastroenterol..

[22] Khan I., Ur Rehman A. (2005). Application of Alvarado scoring system in diagnosis of acute appendicitis. J. Ayub Med. Coll. Abbottabad.

[23] Memon Z.A., Irfan S., Fatima K., Iqbal M.S., Sami W. (2013). Acute appendicitis: diagnostic accuracy of Alvarado scoring system. Asian J. Surg..

[24] Schneider C., Kharbanda A., Bachur R. (2007). Evaluating appendicitis scoring systems using a prospective pediatric cohort. Ann. Emerg. Med..

[25] Ohle R., O'Reilly F., O'Brien K.K. (2011). The Alvarado score for predicting acute appendicitis: a systematic review. BMC Med..

[26] Di Saverio S., Birindelli A., Kelly M.D. (2016). WSES Jerusalem guidelines for diagnosis and treatment of acute appendicitis. World J. Emerg. Surg..

[27] Scott A.J., Mason S.E., Arunakirinathan M., Reissis Y., Kinross J.M., Smith J.J. (2015). Risk stratification by the appendicitis inflammatory response score to guide decision-making in patients with suspected appendicitis. Br. J. Surg..

[28] Di Saverio S., Sibilio A., Giorgini E., Biscardi A., Villani S., Coccolini F. (July 2014). The NOTA study (non operative treatment for acute appendicitis): prospective study on the efficacy and safety of antibiotics (amoxicillin and clavulanic acid) for treating patients with right lower quadrant abdominal pain and long-term follow-up of conservatively treated suspected appendicitis. Ann. Surg..

[29] Nanjundaiah N., Mohammed A., Shanbhag V., Ashfaque K., Priya S. (2014). A comparative study of RIPASA score and ALVARADO score in the diagnosis of acute appendicitis. J. Clin. Diagn. Res.: J. Clin. Diagn. Res..

[30] Díaz-Barrientos C.Z., Aquino-González A., Heredia-Montaño M. (April – June 2018). The RIPASA scale for the diagnosis of acute appendicitis: a comparison with the modified Alvarado score. Gastroenterology Magazine of Mexico.

[31] Gholi Mezerji N.M., Rafeie M., Shayan Z., Mosayebi G. (2015). The diagnostic value of surface markers in acute appendicitis; A diagnostic accuracy study. Bull Emerg Trauma.

[32] Imaoka Y., Itamoto T., Takakura Y. (2016). Validity of predictive factors of acute complicated appendicitis. World J. Emerg. Surg..

